# Controlling an Industrial Robot Using a Graphic Tablet in Offline and Online Mode

**DOI:** 10.3390/s21072439

**Published:** 2021-04-01

**Authors:** Wojciech Kaczmarek, Bartłomiej Lotys, Szymon Borys, Dariusz Laskowski, Piotr Lubkowski

**Affiliations:** 1 Faculty of Mechatronics, Armament and Aerospace, Military University of Technology, Kaliskiego 2 Street, 00-908 Warsaw, Poland; wojciech.kaczmarek@wat.edu.pl; 2 IRLASER sp. z o. o., Al. Jana Pawła II 61/211, 01-031 Warsaw, Poland; bartlomiej.lotys@irlaser.pl; 3 Faculty of Electronics, Military University of Technology, Kaliskiego 2 Street, 00-908 Warsaw, Poland; dariusz.laskowski@wat.edu.pl (D.L.); piotr.lubkowski@wat.edu.pl (P.L.)

**Keywords:** industrial robot, packing, graphics tablet, RobotStudio, RAPID, Visual Studio, sketch-based interfaces, human–robot interaction

## Abstract

The article presents the possibility of using a graphics tablet to control an industrial robot. The paper presents elements of software development for offline and online control of a robot. The program for the graphic tablet and the operator interface was developed in C# language in Visual Studio environment, while the program controlling the industrial robot was developed in RAPID language in the RobotStudio environment. Thanks to the development of a digital twin of the real robotic workstation, tests were carried out on the correct functioning of the application in offline mode (without using the real robot). The obtained results were verified in online mode (on a real production station). The developed computer programmes have a modular structure, which makes it possible to easily adapt them to one’s needs. The application allows for changing the parameters of the robot and the parameters of the path drawing. Tests were carried out on the influence of the sampling frequency and the tool diameter on the quality of the reconstructed trajectory of the industrial robot. The results confirmed the correctness of the application. Thanks to the new method of robot programming, it is possible to quickly modify the path by the operator, without the knowledge of robot programming languages. Further research will focus on analyzing the influence of screen resolution and layout scale on the accuracy of trajectory generation.

## 1. Introduction

The high level of automation and robotisation of modern production lines leads to a search for innovative methods of programming and machine control. This is particularly important in the context of industrial robots, whose operating systems and programming languages provide great freedom in the development of advanced control software [1,2,3,4]. The rapid acceleration of technological progress makes it difficult for process line operators to keep up with the exponentially increasing capabilities of the machines they operate. This is particularly relevant for older and middle-aged people, as they find it much harder to keep up with new technologies. Factory managers strive to minimize production costs, and the increased complexity of robotic workstations makes employee training longer and more expensive. A similar situation can be observed in companies responsible for launching new production lines. In this case, the dynamic development of software requires additional effort from the robotic systems integrators, among others in the form of organizing additional courses and trainings for their employees. With more and more robots in factories, the demand for workers with a specific skill profile is increasing. This creates better paid jobs, but also forces operators to develop in a specific direction. The answer to the increasing demands of flexible production are easy-to-use ways of programming robots. Hence, the solution is more effective integration of man and machine [5,6,7,8].

High competition on the global market makes high flexibility and efficiency of the producer the key to success. Meeting the changing needs of customers in the long term, the constant need to keep production at the highest level, and to deliver the highest quality products to the market is only possible with business strategies based on modern technologies [9]. More efficient new generation machines are becoming a key element of competitive advantage for manufacturing plants. It is thanks to them that processes run faster and more efficiently, and possible errors are automatically eliminated. However, it must be remembered that every technology must be supported by human resources. Despite the fact that most operations on modern production lines are performed fully automatically, practice shows that even the most modern machines require constant control, monitoring and operation performed by qualified personnel. Moreover, in case of failure (e.g., damage of a machine, reduction of production speed or quality) or the necessity to change production parameters, employees are required to react immediately and resume production quickly to minimize losses. Therefore, in the era of Industry 4.0, the control and operation of machines is of greatest concern. It is imperative to search for and implement innovative solutions that ensure easy human–machine interaction [10,11,12]. Specialists and researchers analyzing the development of industrial robotics pay special attention to the competences of operators who will be able to meet the requirements of Industry 4.0. Between 1990 and 2000, the main focus of automation was directed towards computer-integrated production and fully automatic machines on automated production lines. In recent years, the focus has shifted to workers, who have to cope with the dynamically changing nature of factories and the constant introduction of new technologies. We are beginning to talk about anthropocentric production principles, where the person is a valuable resource, essential for the continuity of the company’s operations [13]. The evolutionary stages of operators are closely related to the development of technologies used on production lines [14]. Generation 1.0 operators (1700–1960) performed work manually using hand tools. The Operator 2.0 (1960–1970) generation was supported by CAx tools and NC operating systems. Operator 3.0 (1970–2000) was working on production lines with machines, robots and control computers. The 4.0 generation operator, in order to meet the requirements of Industry 4.0, must work in symbiosis with production systems to ensure maximum production efficiency. The qualitative leap that accompanies the fourth industrial revolution results in humans and machines co-creating a production system [15]. In this system, various components can be distinguished:

- Incremental manufacturing (3D printing)—used to produce complete products [16,17,18],
- systems integration—the flow of data and control signals between machines, information systems, people and management systems [19,20,21,22],
- Industrial Internet of Things (IIoT)—the unified integration of statuary and management systems [23,24],
- Cloud computing—making external hardware and software resources available to process production data and perform complex calculations for the production process itself as well as for management systems [25,26,27],
- big data analysis (Big Data)—i.e., the use of large computing powers to collect, store and analyze large amounts of data [28,29],
- augmented and virtual reality (AR and VR) [30,31,32]—technologies that are proposing to move industry to virtual and augmented reality,
- Cyber security—which plays a huge role in cloud computing and analyzing large data sets on external servers [33,34].

There is research worldwide highlighting the importance of human–machine integration in various aspects. An example is a study [10] that highlights the importance of human–machine integration in the context of data acquisition for reliability-related analyses. Already today, Industry 4.0 integrates humans and digitally controlled machines with the Internet and information technologies [35]. On the one hand, the flow of information is carried out at the level of the production process between machines; on the other hand, the extracted process information feeds into business management systems so that it is easier to forecast production performance. According to [14], the automation of production should not only be oriented towards the implementation of new technologies but should take into account the adaptation to new human conditions, thus improving their physical, sensory and cognitive abilities. Many scientific articles indicate that production systems should be designed not to replace humans, but to assist humans and make them more efficient and effective [36].

This paper presents a method of controlling an industrial robot using a graphic tablet in offline and online modes. The proposed solution allows:

- for easy and intuitive control of an industrial robot by operators without specialized training,
- to program movement of the industrial robot in an easy and intuitive way (task-based) by workers without specialized education,
- to make corrections in the control programs without stopping the robot,
- reduce the number of cables by using wireless, touchscreen tablets,
- development and modification of robot’s control programs using a digital twin in an offline mode, which increases a programmer’s safety, reduces costs and speeds up the software development process.

In the study, we decided to check whether the use of a graphics drawing tablet in a robotic cell would allow the operator to easily define the trajectory of the industrial robot’s movement, and what parameters the defined trajectory should have in order for the robot’s movement to be smooth and the trajectory to be reproduced with high accuracy. In other words, the study dealt with the reproduction of a design made on a graphics drawing tablet by an industrial robot. In addition, we investigated whether it is possible to generate a complex robot motion trajectory using a graphics drawing tablet in a workpiece handling application. In this case, the robot’s task was to move the workpieces from the picking point to the storage point along a path drawn on the graphics drawing tablet.

Before testing on the real bench, we conducted tests in the RobotStudio environment using VR technology. To fully reflect the real conditions, we prepared a digital twin of the actual workstation. This approach ensured a accurate representation of reality. The development of the virtual workstation provided great opportunities especially in terms of conducting functional analyses of the developed software.

The use of programming of the robot or modification of its movements by drawing its trajectory using the screen of a graphic tablet can be used in the case of mobile robots, or service robots, where, e.g., it is necessary to change the robot’s trajectory due to a change in environmental conditions. When using a modern interface (e.g., tablet), it is not crucial to know the programming language of the robot and its operating system. However, it is necessary to know the allowed workspace, which will define the robot’s range of motion.

Section 2 contains related work. In Section 3, we have presented the description and principle of operation of the control programs and the design of the test bench. Section 4 contains the results of the tests carried out. In Section 5, we summarized the results of our work.

## 2. Related Work

There is a worldwide effort to develop programming methods for industrial robots [37,38,39]. These methods can be divided into three groups [40]:

- online (non-text-based),
- offline (text-based, including graphical),
- hybrid (a combination of both methods).

Non-text methods, which are the original methods of robot programming, involve the operator “showing” the robot a specific movement, which is then memorised and reproduced by the robot. Within this group, we distinguish between manual programming and teaching, which is currently most commonly used to program specific actions. This type of programming can be carried out in three ways: directly (the operator manually sets the position of the manipulator), using a robot model (the operator first sets certain actions on the model, which are then transferred by the software to the target robot) and, indirectly, most often using a teach pendant [41,42]. All of the above methods use one of two types of motion: Point-To-Point (PTP), in which the robot memorises successive positions of the trajectory and then moves between them, or Continuous Path (CP), in which the operator moves the manipulator tip along a given trajectory and the robot then accurately reproduces it.

The advantages of non-texting methods include short programming time, but only for simple programs, no or low hardware requirements, and precise execution of learned tasks by the robot. The biggest disadvantages of programming with this method are the need to use the robot, which often involves stopping production, and the long time to design more complex activities [40].

The disadvantages of online programming make text-based methods of programming industrial robots increasingly popular. They allow software development for robots in virtual environments. Thanks to this solution, any software modifications to a robot already working on the production line do not require stopping production. Advanced programming tools facilitate the programming of complex, complicated programmes and at the same time ensure the creation of programme documentation [43]. The disadvantage of offline methods is the need for additional position calibration, as there is a significant risk of incorrect position determination [44,45]. Furthermore, there is often a need for prior verification of the program under real working conditions.

Industrial robot companies and researchers are improving existing programming environments with additional features or creating new environments. The market analysis shows that the world leaders in industrial robotics introduce several major improvements to their environments each year (e.g., ABB’s RobotStudio [46], FANUC’s ROBOGUIDE [47]) or purchase third party solutions e.g., the latest version of Kuka SimPro [48] was purchased from Visual Components [49]. The main advantages of such environments are: comprehensiveness of programming functions, advanced component libraries, implemented virtual robot controllers and the possibility of programming robots both online and offline (e.g., RobotStudio, RT ToolBox3 [50]). However, the main disadvantages include the ability to program robots from a single company and the high price. Users have the possibility to create additional functionalities for them, which support engineers while programming robots, e.g., in the case of RobotStudio environment additional software modules, can be created in VisualStudio and programming languages C# and Visual Basic [51,52].

Another developing trend is environments that enable Generic Robot Programming (GRP) [53]-Robot Operating System (ROS) [54], RoboDK [55], Drag&Bot [56], and RAZER [57]. The main feature of this type of environments is the ability to write control programs for robots of different manufacturers, in one programming language. GRPs have built-in mechanisms that allow for translating the “universal language” into code understandable for particular robots of a given manufacturer. The biggest advantage of GRP is the possibility to create software in a modern programming language (e.g., Python for ROS or in RoboDK). It should be noted that modern programming languages such as Python, C#, and JAVA have advanced libraries that support programmers. The main disadvantage of this type of environment is the lack of virtual controllers, which are available in dedicated environments (e.g., RobotStudio, ROBOGUIDE, RT ToolBox 3, KUKA.SIM Pro). For GRP, it is therefore necessary to develop controllers or code generators which is a very work-intensive and costly activity and therefore such solutions can be very expensive. The most well-known and versatile environment is ROS, but using it requires the programmer to be very familiar with LINUX and is user-unfriendly, which can be a handicap for integrators, engineers and operators who are often not programmers. In addition, this type of environment does not provide a full representation of programming languages, an example being RoboDK, which does not support conditional statements (e.g., if, then, else).

Highly advanced development environments (Process Simulate [58], Delmia [59], Visual Components [49]) are also available on the market, which integrate CAD systems with advanced offline programming of industrial robots. Their advantage is the ability to program robots from different manufacturers in a single project. However, in this case, the prices of the environments are very high and full simulations are possible after buying additional virtual controllers for individual robots.

Increasing demands on operators necessitate work on new methods of machine programming and control. As already mentioned, operating systems and robot programming languages provide great freedom to create advanced control software. Traditional robot programming using dedicated teaching panels is slowly becoming rare and is being replaced by programming using computers and other systems (e.g., vision systems). Forms of human–machine communication that are similar to those used by humans on a daily basis are being sought. One example is the use of gestures. This way of communication is already used today to control TV sets, household appliances (e.g., cleaning robots), transport vehicles (mobile robots—[60]) and industrial robots [61]. In solutions related to gesture-based robot control, many researchers use the KINECT sensor [51,62,63,64].

The possibility of controlling robots using different types of manipulators (teleoperators) is also being investigated. This research is mainly developed to use robots in medicine, where a properly configured system allows for performing surgeries [65]. A very difficult issue is to control systems with a high number of degrees of freedom [66]. In addition, many years of research are required to confirm the reliability of the proposed solutions in medical applications. Another important problem is collision avoidance and detection by the robot during operation. In the case of online programming, the programmer is able to correct the robot’s path in real time. When working offline, it is useful to use collision detection. Due to the importance of the problem, work is also being carried out to automatically avoid collisions and retreat from problematic points [67,68,69].

## 3. Materials and Methods

**Figure 1:**
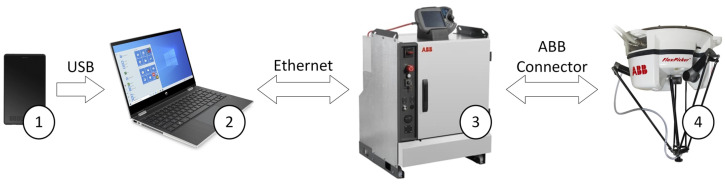
General station view: 1—Huion graphics tablet, 2—PC, 3—IRC5 controller, 4—IRB 360 industrial robot.

The integration of the devices forming the stand (IRB 360 FlexPicker robot, graphic tablet, computer) involved the development of an application for operating the tablet and carrying out the process of controlling the industrial robot, ensuring communication between the devices and allowing interaction with the operator (Figure 1).

The Huion graphics tablet used consists of an active working area and a zone of function keys (working area—221 × 138 mm, resolution—5080 LPI, sampling rate—233 RPS). We used a stylus to operate it. Using this solution allowed us to significantly increase the accuracy of the drawn tracks on a computer without a touch screen. Thanks to this, we obtained a high accuracy of mapping drawn trajectories and geometric patterns. At the workstation, we used a computer with Windows 10 operating system to control an industrial robot using a graphics tablet. Thanks to the control programs developed, the computer provided support for the graphics tablet (programs developed in C# language in Visual Studio) and communication with the robot controller (programs developed in RAPID language in RobotStudio and C# language in Visual Studio). PC SDK 6.07 libraries (ABB.Robotics.Controllers) were used to operate the IRB 360 robot. A key issue for this project was to properly define the robot’s parameters and the points that form the paths, in such a way that they could be read and processed by the IRC5 robot controller. We developed an algorithm that formats the positions from the graphics panel and writes them to a position table—following the Rapid language position creation standard. Additionally, we have developed control programs that allows:

- connection to a selected robot (connected to a local Ethernet network),
- switching between robot modes between transferring workpieces and drawing the corresponding pattern,
- controlling the working parameters of the robot and its start-up.

Thanks to this solution, we can control ABB industrial robots connected to an Ethernet network, in which a PC with a graphics tablet and a tablet running Windows 10 (additional option for creating robot motion trajectories) are also working.

### 3.1. System Design

**Figure 2:**
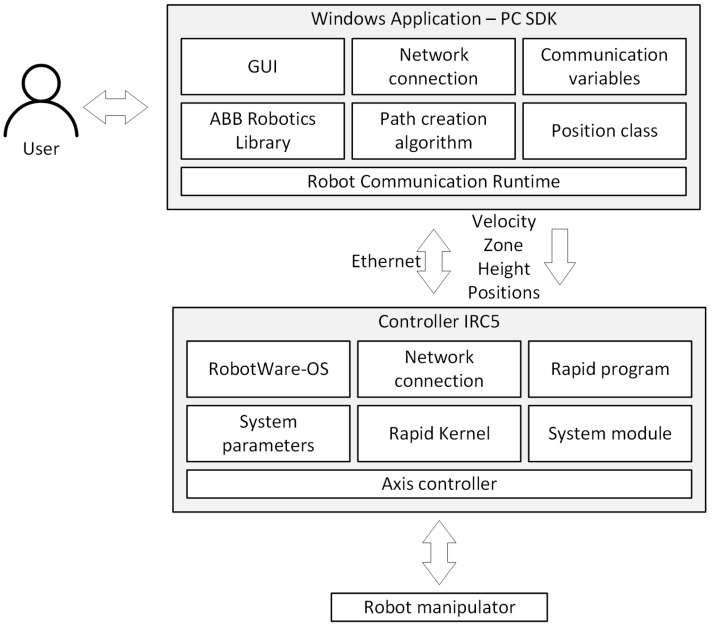
High-level architectural diagram of the application’s communication with the robot controller.

In Visual Studio, we developed a control application in C# language, which is responsible for the configuration of the transferred data and communication between the PC unit and the robot controller (Figure 2). In addition, we developed a graphical user interface for it, allowing the robot to be operated freely without having to interfere with the programme code. To control the robot, we used a Huion graphic tablet compatible with the Windows 10 operating system.

The application communicates with the robot via Ethernet using the TCP/IP data transmission protocol. During its development, we used the ABB.Robotics.Controllers and System.Net libraries. The application using the ABB libraries sends data to the Robot Communication Run Time module, which is responsible for synchronizing the code and sending data in both directions. The former library allows direct access to the robot controller before running its program. This makes it possible to retrieve and set the values of various data and parameters of the robot’s control program from the level of the computer application, e.g., start points of the robot program, speed of the manipulator, accuracy of path mapping. The second library helps to control the robot while it is executing its programme. It gives the possibility to send and receive signals, e.g., send control signals and receive the current position of the robot manipulator. We developed the control program for the ABB IRB 360 FlexPicker robot in the RAPID language in ABB RobotStudio.

The main functionality of the developed application is the ability to create a pattern on the graphic panel and save the position of the moving cursor to the position list. For this purpose, we used the program control events such as StylusDown, StylusMove, StylusUp. These events reflect the drawing process:

- the StylusDown event is used for when the pen is placed on the page,
- the StylusMove event is used for moving the pen and drawing the pattern,
- the StylusUp event is used for when the pen is lifted from the page.

**Figure 3:**
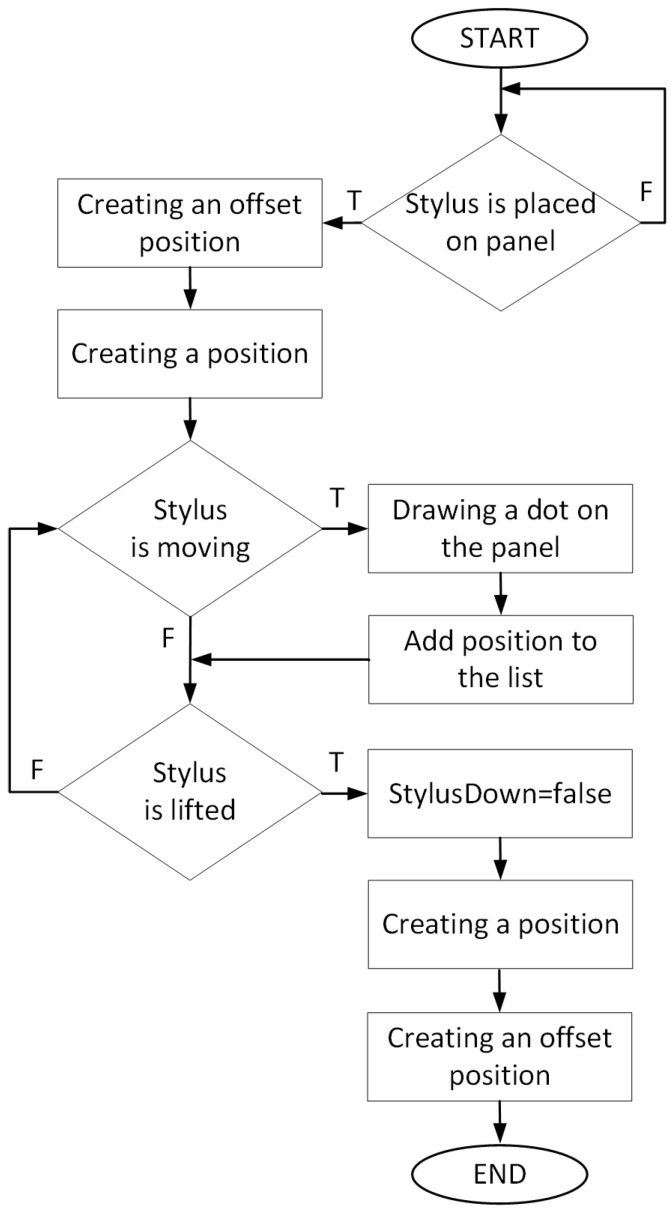
Algorithm for creating patterns and item list.

Such a solution ensured easy programming of the industrial robot since the robot’s movement trajectory was divided into three stages (pick, move, place). The adopted programming principle can be directly adapted to the programming of robots on production lines, where the above-mentioned stages are characterised by different parameters (velocity, acceleration, deacceleration, accuracy). The algorithm for creating the pattern on the graphic panel and the list of positions is shown in Figure 3.

In addition to a full-featured application that allows you to control an industrial robot using a graphics tablet, we developed an intuitive operator interface that allows you to easily start up and control an industrial robot. We used Microsoft Visual Studio 2017 and the C# language for this purpose. The application allows the user to control a robot, selected from a list of available robots on the Ethernet network. The main task of the application is to allow the operator to control the industrial robot based on the paths drawn on the graphic panel. The mapping of the paths by the robot is performed with respect to the set motion parameters of the robot, such as velocity, zone, acceleration and deceleration.

**Figure 4:**
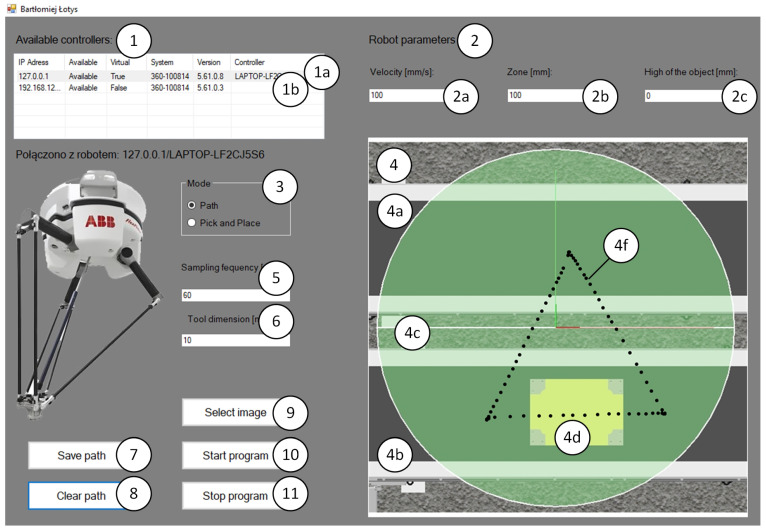
General GUI view with generated movement trajectory, 1—available controllers, 1a—virtual controller, 1b—real controller, 2—robot parameters, 2a—velocity, 2b—zone, 2c—object hight, 3—mode, 4—layout of station, 4a—input conveyor, 4b—output conveyor, 4c—robot workspace, 4b—obstacles, 4f—generated path, 5—sampling frequency, 6— tool dimension, 7—save path button, 8—clear path button, 9—select image button, 10—start program button, 11—stop program button, 12—information about selected controller, 13—general view of the robot.

The application developed in VisualStudio in C# language allows for generating paths for a delta robot. The application uses the parameters of ABB’s IRB 360 robot. It is equipped with a graphical user panel (Figure 4) that:

- Displays information about the robot controllers present in the Ethernet network (real and virtual controllers—Figure 4 (1)). After selecting a robot, the controller with which the connection has been established is displayed under the window of available controllers (Figure 4 (12))
- Connect to a selected controller (real or virtual) and take control over the selected controller.
- Cooperate with a program developed in RAPID language that controls an industrial robot. The main task of the application is to be able to change the robot’s trajectory, both the position of individual points forming the trajectory and the robot’s motion parameters (TCP speeds, zones of passage through points, heights of objects).
- Allows selecting the operation mode. Two operation modes have been prepared (Figure 4 (3)):
  - Path—used to generate complex motion trajectories.
  - Pick and Place—used to modify trajectories carried out during the transfer of products.
- Allows for defining selected parameters of robot’s movement (TCP speeds, zones of passage through points, heights of objects—Figure 4 (2)).
- Define the sampling frequency (Figure 4 (5)). The application allows for creating continuous paths. This enables the user to create complex trajectories with different shapes.
- Allows definition of tool dimensions (Figure 4 (6)). When generating a path, the operator has the possibility to define the dimensions of the robot tool (e.g., gripper) in the form of the diameter of the inserted points, which allows for generating trajectories between obstacles located at the workstation and prevents the robot from causing collisions during the process. The operator controls how obstacles are avoided during the creation of the trajectory.
- Allows selection of the station layout (Figure 4 (4,9)) and importing it to the developed application. The user has the option to select a station view with the robot’s working range marked (Figure 4 (4c)), which allows the generation of trajectories (Figure 4 (4f)) in the area available to the robot. If an attempt is made to start the robot with an uploaded trajectory that is completely or partially outside the robot’s area, the robot stops and displays an error of not being able to reach the set position.
- Saves the generated path, which can be reused several times (Figure 4 (7)).
- Deletes the path in case it needs to be modified (Figure 4 (8)).
- Starts and stops the control program (selected in Available controllers window) of the robot (Figure 4 (10,11)).

### 3.2. Test Stand

**Figure 5:**
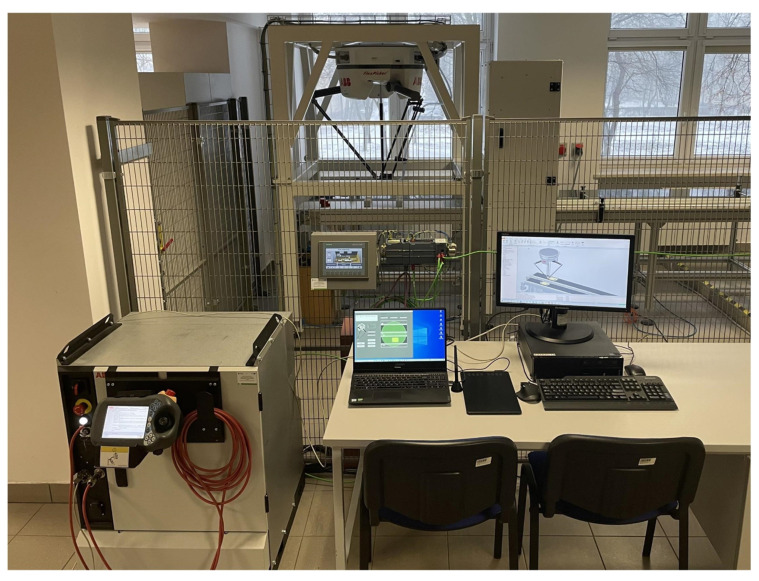
General view of the FlexPicker station in Robotics Laboratory.

Tests of the application correctness were carried out on a test stand in the Robotics Laboratory of the Military University of Technology (Figure 5). The workstation consists of:

- ABB IRB360 FlexPicker robot with IRC5 controller,
- graphic tablet,
- personal computer.

The IRB 360 family includes variants with payloads of 1 kg, 3 kg, 6 kg and 8 kg and reaches of 800 mm, 1130 mm and 1600 mm—meaning that there is an IRB 360 for almost every need. We used a robot with a 1 kg payload and the 1130 mm work area.

To control a manipulator, knowledge of forward and inverse kinematics is required [70]. The robot in question is a delta robot with four degrees of freedom and consists of three identical arms connected in parallel. In this case, the orientation of the robot remains constant, hence it is necessary to control the three axes x,y,z. The side length of the base platform (B) is $s_{B}$, the side length of moving platform (P) is $s_{P}$. The joint variables are:

(1) $$\Theta ={\left\{\theta_{1}\;\theta_{2}\;\theta_{3}\right\}}^{T}$$

where $\theta_{1},\;\theta_{2},\;\theta_{3}$—actuated revolute joint angles.

The Cartesian variables are:

(2) $${}^{B}P_{P}={\left\{x\;y\;z\right\}}^{T}$$

where x, y, z—Cartesian coordinates.

**Figure 6:**
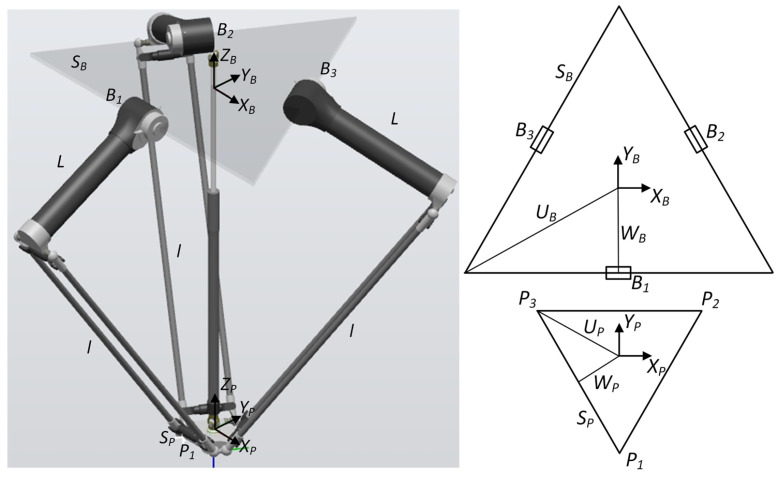
Delta manipulator schematic.

A schematic of the robot is shown in Figure 6.

Forward kinematics are described by the following equations. Three absolute vector knee points are given as:

(3) $${}^{B}A_{i}{=}^{B}B_{i}{+}^{B}L_{i}\;i=1,\;2,\;3$$

where

(4) BB1=0−wB01BB2=32wB12wB01BB3=−32wB12wB0

(5) BL1=0−Lcosθ1−Lsinθ11BL2=32Lcosθ212Lcosθ2−Lsinθ21BL3=−32Lcosθ312Lcosθ3−Lsinθ3

Three virtual sphere centers are given as:

(6) $${}^{B}A_{i\nu }{=}^{B}A_{i}{-}^{P}P_{i}\;i=1,\;2,\;3$$

where

(7) BA1ν=0−wB−Lcosθ1+up−Lsinθ11BA2ν=32(wB+Lcosθ2)−sP212(wB+Lcosθ2)−wP−Lsinθ21BA3ν=−32(wB+Lcosθ3)−sP212(wB+Lcosθ3)−wP−Lsinθ3

(8) PP1=0−uP01PP2=sP2wP01PP3=−sP2wP0

$w_{B}$—planar distance from {0} to near base side,
*L*—upper legs length,
$u_{P}$—planar distance from {P} to a platform vertex,
$s_{P}$—platform equilateral triangle side,
$w_{P}$—planar distance from {P} to near platform side.

Forward kinematics unknown point (${}^{B}P_{P}$) is the intersection of the three known spheres:

(9) (1BA1ν,l)(1BA2ν,l)(1BA1ν,l)

where *l* is lower legs parallelogram length.

Inverse kinematics are described by the following equations:

(10) $$E_{i}\cos\theta_{i}+F_{i}\sin\theta_{i}+G_{i}=0\;i=1,\;2,\;3$$

where

(11) $$E_{1}=2L\left(y+a\right);\;E_{2}=-L\left(\sqrt{3}\left(x+b\right)+y+c\right);\;E_{3}=L\left(\sqrt{3}\left(x-b\right)-y-c\right)$$

(12) $$F_{1}=2zL;\;F_{2}=2zL;\;F_{3}=2zL$$

(13) $$\begin{array}{c} G_{1}=x^{2}+y^{2}+z^{2}+a^{2}+L^{2}+2ya-l^{2};\;G_{2}=x^{2}+y^{2}+z^{2}+b^{2}+c^{2}+L^{2}+2\left(xb+yc\right)-l^{2}\\ G_{3}=x^{2}+y^{2}+z^{2}+b^{2}+c^{2}+L^{2}+2\left(-xb+yc\right)-l^{2} \end{array}$$

(14) $$a=w_{B}-u_{P};\;b=\frac{s_{P}}{2}-\frac{\sqrt{3}}{2}w_{B};\;c=w_{P}-\frac{w_{B}}{2}$$

Required actuated revolute joint angles can be calculated from:

(15) $$\theta_{i}=2\tan^{-1}\left(\frac{-F_{i}\pm \sqrt{E_{i}^{2}+F_{i}^{2}-G_{i}^{2}}}{G_{i}-E_{i}}\right)$$

**Figure 7:**
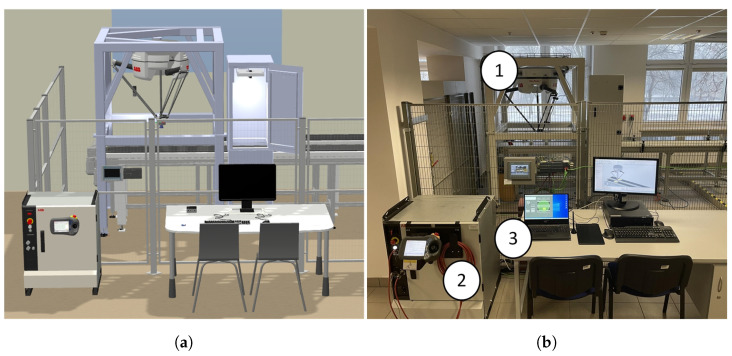
General station view: (**a**) Digital Twin; (**b**) real station, 1—IRB 360 robot, 2—IRC5 controller, 3—Windows based PC.

One of our objectives was to develop a digital twin of the real robotic station (Figure 7). As part of the work carried out, we prepared models of components (devices, mechanisms and structures) included in the real workstation. These models have been transformed into RobotStudio environment libraries. Using the functions available in RobotStudio, we created a virtual layout, which is a 100% mapping of the real station.

Next, a backup of the actual robot was used to create a digital twin of the system. In the RobotStudio environment, a robot system was generated from the backup, which runs on a virtual controller. This enabled the real robot controller to be replicated with almost 100% accuracy (according to the producer). This means that the robot model in the virtual environment has the functionality and features of the real device. As a result, it is possible to program the robot model taking into account all the capabilities and limitations of the real robot, and the results obtained during the simulation are reliable. This made it possible to create, modify and test software safely without having to work online with the real robot. This has been confirmed during tests where, among other things, the results obtained in simulations were verified against the results obtained in the real station. The combination of devices in both cases was the same. During tests with the virtual station, a second computer with a virtual station developed in RobotStudio was connected in place of the robot controller. The prepared workstation can be easily used for further software development.

## 4. Evaluation

**Figure 8:**
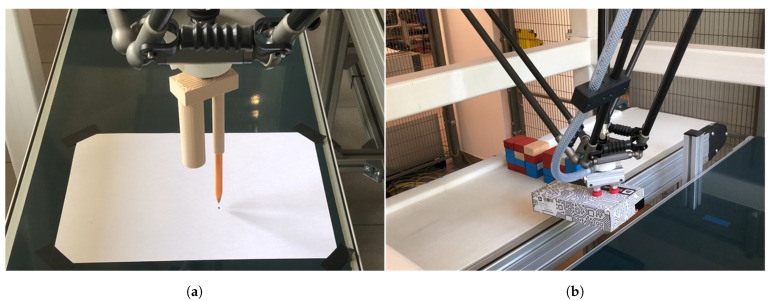
General view of IRB360 robot with: (**a**) pen; (**b**) suction gripper carrying a detail.

We have tested the application in RobotStudio (digital twin), and on a real packaging station (Figure 8). The developed digital twin allowed us to verify the correctness of the developed control programmes and the correctness of data exchange between devices. In the RobotStudio environment, we observed the impact of changes in the robot’s motion parameters on the reproduction of the motion path and the correctness of I/O signals. Testing the application in offline mode eliminated the risk of a collision between the robot and surrounding elements.

### 4.1. Test 1

**Figure 9:**
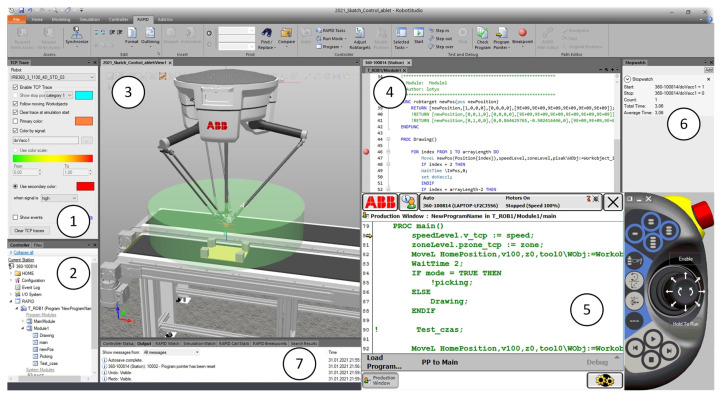
General view of RobotStudio environment, 1—TCP Trace options, 2—Project Tree, 3—Station Layout, 4—Program Editor, 5—Virtual FlexPendant, 6—Stopwatch options, 7—Output window.

The first study consisted of generating a robot trajectory in a developed application using a graphics tablet and checking its correct execution by a digital twin (Figure 9) of a robotic sorting and packaging station using ABB’s IRB 360 robot.

Test 1 used the following application settings:

- Velocity 200 mm/s,
- Zone 0 mm,
- Hight of object 0 mm,
- Mode Path,
- Sampling frequency 16 Hz,
- Tool dimension 10–100 mm.

The test involved verification of the correctness of path clearance at different tool dimensions. Firstly, a top view of the virtual workstation with the robot workspace was taken and imported to the software. Then, the robot motion trajectory was generated for given Tool Dimension values, with selected shapes, and the robot operation parameters were set.

**Figure 10:**
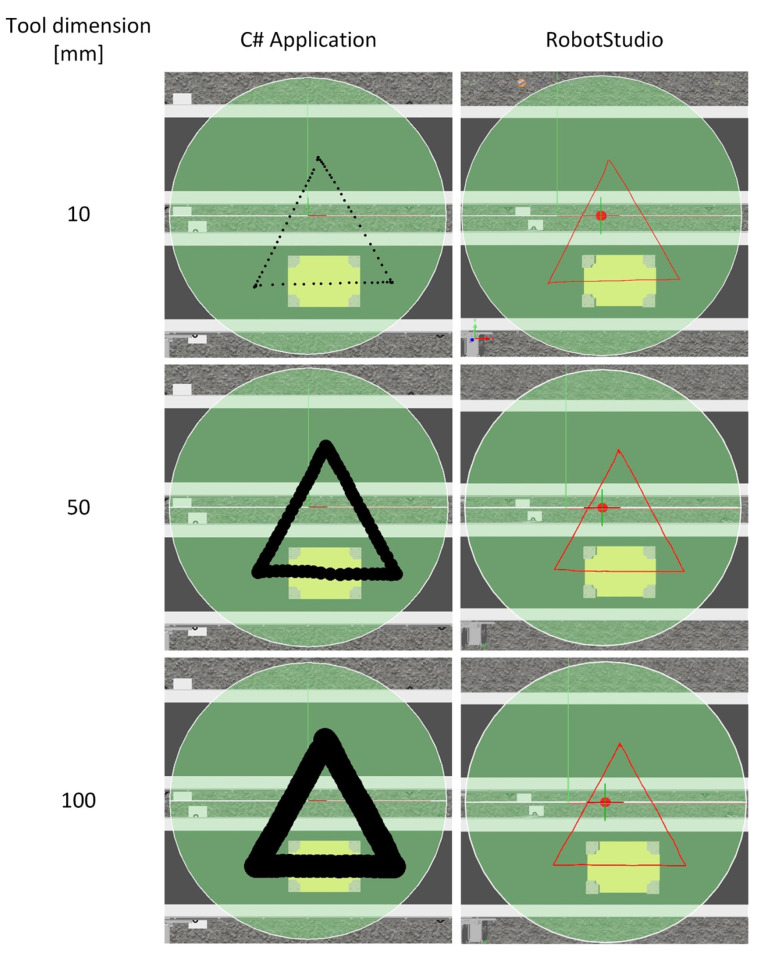
Work areas generated in the Application and RobotStudio environment.

The tests carried out confirmed the correct functionality of the original application. The paths drawn in the application were generated and exported to the industrial robot controller. As shown in Figure 10, the robot correctly and accurately reproduced the drawn path, which can be seen in the figures in the RobotStudio column. The trajectory presented was plotted using the TCP Trace tool. The correctness of the realization of the path by the robot was verified by comparing the coordinates generated by the application, with the coordinates of the realized trajectory. The evaluation of the path realization was based on the coordinates of the triangle tips, which coincided in both cases.

Figure 10 shows examples of the generated trajectory in the developed application and views of the plotted path by the virtual twin in the RobotStudio environment.

Thanks to the possibility of defining the tool dimension, the operator is able to generate a path taking into account the tool parameters. This allows for avoiding collisions with obstacles present in the workspace.

### 4.2. Test 2

The second test consisted of verifying the correct representation of the robot’s motion trajectory for different sampling times.

Test 2 used the following application settings:

- Velocity 200 mm/s,
- Zone 0 mm,
- Hight of object 0 mm,
- Mode Path,
- Sampling frequency 1–33 Hz,
- Tool dimension 10 mm.

**Figure 11:**
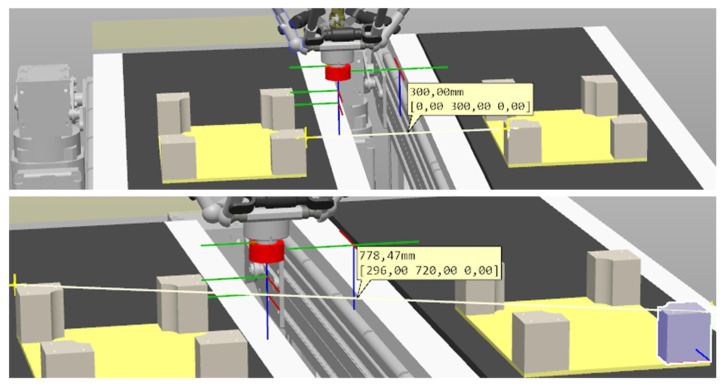
Station layout in RobotStudio.

The test was performed for the movement of the robot between the points shown in Figure 11. To perform the test, trays with boxes were placed on both feeders. The location of the objects is shown in the figure above. The distance between the trays was 300 mm, and the resulting distance between the selected points was 778.47 mm. The start and end points were chosen so that the robot movement is performed in two axes (X and Y)—the coordinates of the points are stored in the defined Workobject coordinate system.

Table 1 lists the coordinates of the start and end points of the generated trajectory (for different sampling times) in the form of coordinates sent to the robot.

The times were measured using the Stopwatch function in RobotStudio. For each case, 10 measurements were made. The time measurements were controlled by the signal doVacc1. The measurements were made for the path shown in Figure 11. The results for each test are shown in the following table.

**Figure 12:**
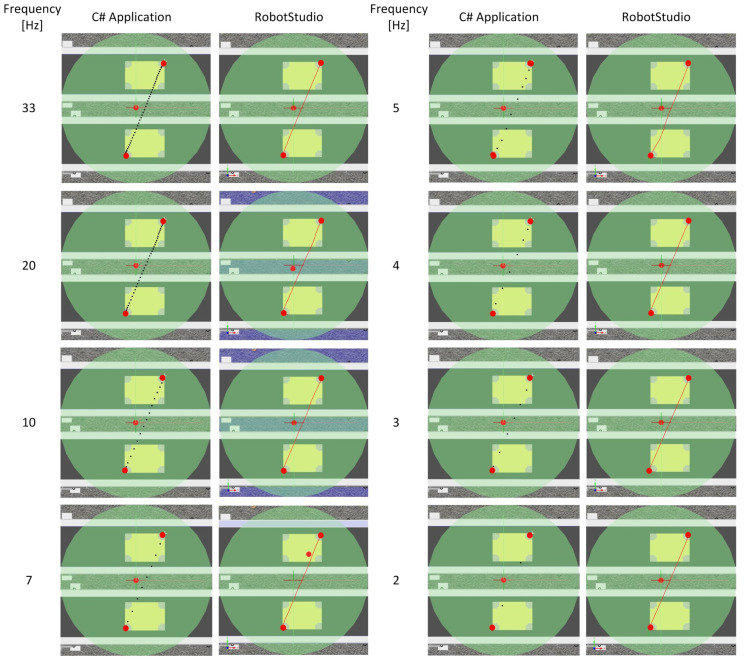
Test 2 results.

The results presented in Table 1 and Figure 12 confirm the correctness of the application. Figure 12 presents the paths drawn in the application and exported to the robot controller. In order to verify the cycle time for different sampling rates, each test was performed 10 times. The set sampling time does not significantly affect the obtained cycle time of the robot, the average time for the performed tests were 3.96 s. The largest deviation from the average was 4.2%. It should be noted that the cycle time is affected by the coordinates of the start and end points plotted by the operator. As can be seen in Table 1, the greatest differences in coordinates for the start point—X = 10, Y = 6, while for the end point—X = 10, Y = 16. Since the movement was performed only on the xy plane, the Z coordinate remained constant. The trajectories were generated in a 565 × 565 pixel window which affected the results obtained. Increasing the precision of the drawing is possible by increasing the active area and decreasing the scale of the station. Considering the influence of the pen drawing speed, the possibility to offset it by the sampling frequency seems to be a necessary feature of such an application.

### 4.3. Test 3

The idea of generating trajectories by the operator using a graphic panel could be used in case of a necessity to quickly modify the trajectory of movement. A great advantage of the proposed solution is that the operator does not need to know the robot’s programming language. It should be noted that robots offered on the global market by individual companies have dedicated programming languages (e.g., RAPID, KAREL, KRL, AS, Melfa Basic).

**Figure 13:**
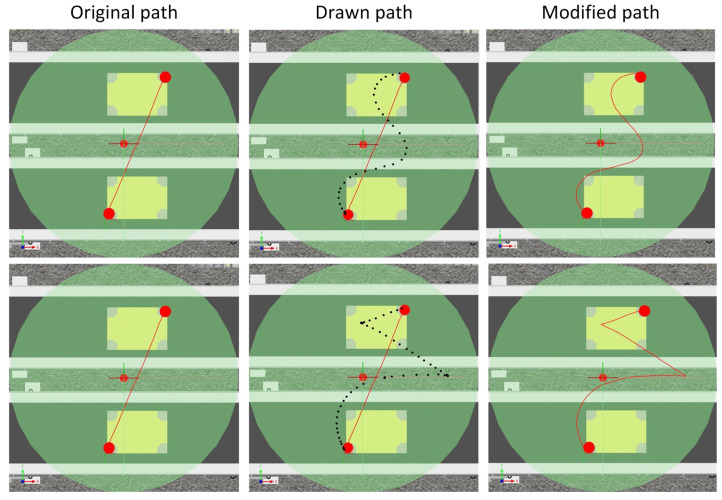
Test 3 results.

Figure 13 shows two examples of modifying the robot’s trajectory using the prepared application. As can be seen in the individual images, the operator was able to change its nature without knowledge of the RAPID programming language.

## 5. Conclusions

The application developed in C# allows the configuration of the data sent to the robot and provides communication between the computer and the industrial robot. The proposed user interface, in combination with a graphic tablet, allows the control of the robot and the easy creation of complex robot trajectories in a designated workspace. Moreover, it is possible to operate the application using a computer mouse and a touch screen. The functionality of the application includes the possibility to work with multiple controllers (both virtual and real). The operator has the possibility to enter the robot motion parameters, select the operation mode (pick and place, path), import the layout of the robot station and generate the drawn trajectory of the industrial robot motion as well as starting and stopping the manipulator.

The work involved developing a digital twin of a real production cell based on ABB’s IRB360 robot. The use of this technology significantly shortened the process of software development and testing. It allowed safe testing of the application and analysis of the impact of the generated trajectories on the robot’s operation. The results obtained during tests on a virtual and real workstation indicate that a graphic tablet can be a helpful and easy-to-use tool on robotized production lines. This could be of particular importance in the era of personalization of automatically manufactured products and the increasing need for close human–machine collaboration. Transferring complex trajectories drawn on a graphics tablet is not an easy task due to the limited number of degrees of freedom of the robot, especially if the orientation of the effector is taken into account. In addition, depending on the task at hand, it is necessary to select an appropriate sampling rate of the drawn trajectory. Adequate selection of the sampling frequency should ensure smooth movements of the robot while maintaining the assumed degree of accuracy of the trajectory mapping. The tests carried out show that the operator must be skilled, and inaccuracies in this performance can significantly affect the achieved performance of the station.

While conducting the tests, it was noticed that, for the proposed method of generating the robot’s motion trajectory, special attention should be paid to the accuracy of determining the start and end points. In the case of graphic tablets and touch devices, the problem is the resolution and scale of the imported layout. With higher resolution devices, the user has the opportunity to increase the accuracy of path generation. The same effect will certainly be exerted by the scale of the station layout imported into the application—the larger the scale, the greater the precision. The virtual station will be used in the future to develop software for programming industrial robots using mobile devices with Windows and Android operating systems. In the next step, tests will be carried out to analyze the influence of screen resolution and layout scale on the accuracy of trajectory generation.

## Figures and Tables

**Table 1:** Test 2 results.

Sample	Frequency [Hz]	Start Point [mm]			End Point [mm]			Average Time [s]
		x	y	z	x	y	z	
1	33	199	−343	0	−89	357	0	4036
2	20	209	−339	0	−85	353	0	4132
3	10	205	−343	0	−87	357	0	402
4	7	203	−343	0	−87	361	0	3912
5	5	207	−343	0	−85	361	0	3908
6	4	201	−337	0	−89	367	0	3904
7	3	203	−341	0	−95	367	0	3924
8	2	197	−339	0	−87	351	0	3842
